# Disease properties, geography, and mitigation strategies in a simulation spread of rinderpest across the United States

**DOI:** 10.1186/1297-9716-42-55

**Published:** 2011-03-24

**Authors:** Carrie Manore, Benjamin McMahon, Jeanne Fair, James M Hyman, Mac Brown, Montiago LaBute

**Affiliations:** 1 Department of Mathematics, Oregon State University, Corvallis, OR 97331, USA; 2 Los Alamos National Laboratory, Theoretical Biology and Biophysics, Los Alamos, NM 87545, USA; 3 Los Alamos National Laboratory, Biosecurity and Public Health, Mailstop M888, Los Alamos, NM 87545s, USA; 4 Los Alamos National Laboratory, Applied Mathematics and Plasma Physics, Mailstop B284, Los Alamos, NM 87545 and Tulane University, New Orleans, LA 70118, USA; 5 Los Alamos National Laboratory, System Engineering and Integration, Mailstop K551, Los Alamos, NM 87545, USA

## Abstract

For the past decade, the Food and Agriculture Organization of the United Nations has been working toward eradicating rinderpest through vaccination and intense surveillance by 2012. Because of the potential severity of a rinderpest epidemic, it is prudent to prepare for an unexpected outbreak in animal populations. There is no immunity to the disease among the livestock or wildlife in the United States (US). If rinderpest were to emerge in the US, the loss in livestock could be devastating. We predict the potential spread of rinderpest using a two-stage model for the spread of a multi-host infectious disease among agricultural animals in the US. The model incorporates large-scale interactions among US counties and the small-scale dynamics of disease spread within a county. The model epidemic was seeded in 16 locations and there was a strong dependence of the overall epidemic size on the starting location. The epidemics were classified according to overall size into small epidemics of 100 to 300 animals (failed epidemics), epidemics infecting 3 000 to 30 000 animals (medium epidemics), and the large epidemics infecting around one million beef cattle. The size of the rinderpest epidemics were directly related to the origin of the disease and whether or not the disease moved into certain key counties in high-livestock-density areas of the US. The epidemic size also depended upon response time and effectiveness of movement controls.

## Introduction

Animal diseases, such as foot-and-mouth disease and avian influenza, are increasingly important in world economics, national security, and biodiversity. Introduction of an exotic livestock disease to the United States (US) either by natural or anthropogenic means could have serious economic and public health consequences. Direct costs due to recent outbreaks of mad cow disease and foot-and-mouth disease in the United Kingdom cost billions of dollars in death of animals, culling, and vaccination. Although direct costs can be enormous, indirect costs such as loss in livestock exports are often much greater. In addition to economic loss, animal diseases are often a human public health threat. Many animal diseases (e.g., avian flu, tularemia, monkeypox) are zoonotic and can be spread from animals to humans.

To help prepare for the possibility of a serious animal disease epidemic, we created a spatially explicit stochastic model for multi-host animal diseases to better understand their spread in the US. The model uses county-level data and between-state animal transportation rates to capture both the intra-county and inter-county behavior of an epidemic. The model is flexible and can be used to simulate many types of animal diseases among various animal groups (poultry, cattle, pigs, etc.) while incorporating surveillance and response strategies.

Rinderpest is a virus closely related to human measles and canine distemper that affects cloven-hoofed animals such as cows, pigs, sheep, and wild or domestic buffalo [1,2]. This virus can cause high morbidity and mortality in naive populations, is highly transmissible and has a long history of devastating livestock herds and wildlife in Europe, Asia, and Africa [2,3]. During World War II, vaccinations for rinderpest were developed and produced in response to a possible threat of rinderpest introduced to the US [4].

Rinderpest has a fairly short incubation period of 4 to 5 days followed by 1 to 2 weeks of clinical signs, including fever, loss of appetite, lesions, diarrhea, dehydration, and death. Clinical signs can continue for many weeks as animals recovering from the acute phase suffer debility, secondary infection e.g. skin disease, eye pathology and other manifestations. In its most virulent form and with a high density population of naïve animals, rinderpest is a fast-moving disease that requires a large number of susceptible animals to persist [5,6]. There are avirulent strains of rinderpest that have occurred in many different situations, but we will focus on virulent and/or rapidly spreading strains. Mariner et al. [2] estimated the reproductive number of the more virulent lineage of rinderpest to be 4.4 and 1.2 for the less virulent lineage.

A relatively mild form of rinderpest endemic to cattle can have devastating effects on wildlife populations and vice versa. Domestic cattle and wild or domestic buffalo have the highest death rates due to rinderpest but it also affects sheep, goats, pigs, and many wildlife species [7]. Additionally, wildlife populations may be an important source of re-infection of rinderpest [8]. European bison and deer were susceptible to rinderpest with high mortality rates. White-tailed deer have also been infected experimentally, so it is likely they and other wildlife species could be a factor in the spread of rinderpest in the United States. For the past decade, the Food and Agriculture Organization of the United Nations has been working on eradicating the disease through vaccination and intense surveillance and was officially considered eradicated in October, 2010 [9]. Rinderpest virus was last confirmed in wild buffalo in Kenya in 2001-2002 and there is no confirmed case or serological evidence of circulation of virus amongst wildlife since then. Equivocal serology from cattle due to rinderpest has not been confirmed in any location or livestock population within the declared infection zone of the Somali ecosystem of East Africa since that period and all vaccination has ceased since 2003 [9,10].

However, due to severity of rinderpest epidemics--and like smallpox-- it will remain a disease to research if it were to infect animal populations outside the laboratory. If rinderpest were to emerge in the US, the loss in livestock would likely be devastating. Rinderpest has never been detected in North America so there is no immunity to the disease among our livestock or wildlife. Historically, introduction into näive herds causes high death rates [11]. In the 1890s, the effects on cattle herds in eastern Africa and large portions of sheep, goat, and ungulate wildlife populations were severe, changing the distribution of animals in many regions of Africa. Consequences of this epidemic for people living in the area included famine for some pastoral groups in sub-Saharan Africa, including the Maasai. It was also a catalyst for the re-emergence of human diseases such as sleeping sickness, which were temporarily absent due to the loss of tsetse fly hosts in regions of Africa caused by rinderpest mortality [6,11,12]. If rinderpest entered the US, it could be devastating to animal agriculture, wildlife, and the economy. To investigate effective responses to an introduction of rinderpest to the US, we have adapted our spatial epidemiology model specifically to the behavior of primary hosts of rinderpest.

James and Rossiter [6], Lefèvre et al. [1], and Mariner et al. [2] have previously developed mathematical models for the spread of rinderpest in Africa. All three incorporate different vaccination programs and stochasticity to explore the spread of rinderpest in cattle herds within parts of Africa where the disease is either endemic or has been present in the past. Their models do not include multiple hosts or spatial heterogeneity, both of which are important to the spread of rinderpest. The models were used for previously exposed or vaccinated herds and some of the parameter values would not be accurate for an epidemic in the US, since rinderpest is an exotic disease for the US and all animals would be immunologically näive. Our model extends and expands the ideas in these models to include multiple mitigation strategies, spatial spread among counties on a network, multiple host categories, and the effects of rinderpest on näive herds.

Our objective was to model a rinderpest outbreak in the US to determine agricultural and veterinary practices that minimize the risk of catastrophic damage from this exotic disease. Using an epidemiological model, we explore the effectiveness of various mitigation strategies such as surveillance, quarantine, vaccination, movement control, and culling, which are incorporated in the model. We determine the sensitivity of the model to these strategies and compare results for different responses in order to minimize risk and damage. For rinderpest, the relevant groups of livestock are sheep, hogs and pigs, dairy cows, cattle on feed, and beef cattle. The mathematical model was used to estimate the extent of spread in, and the relevance of, each of these groups. Because there are no data for rinderpest in the US, our model is useful for creating a plan of action should an outbreak occur.

## Methods

Here we present a two-stage hybrid model of the spread of a multi-host infectious disease among agricultural animals in the US using rinderpest as a case study. The model incorporates large-scale interactions between US counties and the small-scale dynamics of disease spread within a county. The large-scale interactions and spread of disease between counties is stochastic. To model within county dynamics, we analyze a distribution of solutions to deterministic equations (see Section "Intra-county model") with parameters sampled from the ranges in Tables 1, 2, and 3. The model is designed to be as general as possible so that it can be adapted to varying parameter values and situations.

**Table 1:** Model parameter description and disease input ranges used with supportive references

	Parameter description	Baseline	Range	**Ref**.
*ι_i_^I^*	infectivity of species *i *in stage *I*	0.00000023	N/A	[12]
*ι_i_^L^*	infectivity of species *i *in stage *L *(subclinical)	0.000000115	N/A	[8]
*ι_i_^C^*	infectivity of species *i *in stage *C *(carrier infectivity)	0.000000115	N/A	[12]
*s_4_^s^*	susceptibility of susceptible stage feedlot cattle	5.0	N/A	[19-21]
*s_i_^s^*	susceptibility of animals besides feedlot in stage *S*	22.5	N/A	[21]
*r(X)*	1/measure of density of animals in county		N/A	USDA [1]
*a*	constant of proportion for contact rate	5	N/A	N/A
*β_ij_^mn^*	transmission rate from type *j *in stage *n *to type *i *in stage *m*		N/A	[6,17,18,21]
*r_Vs_*	reduced susceptibility of vaccinated susceptible animals	0.5	N/A	[22-24]
*r_Ve_*	reduced infectivity of vaccinated quiescent infected animals	0.5	N/A	[24,25]
*λ_L_*	rate of progression from latent to infectious stage (1/residency time in stage)	1/4.5 days	3-6	[26,27]
*λ_C_*	rate of progression from carrier to recovered	1/698.75 days	120-1277.5	[28]
*λ_I_*	rate of progression from infectious to recovered	1/6 days	4-8	[8,28]

**Table 2:** Model parameter description and disease input ranges used with supportive references

	Parameter description	Baseline	Range	**Ref**.
*λ_Vs_*	rate of progression from vaccinated susceptible to recovered	1/10.5 days	7-14	[28]
*λ_Ve_*	rate of progression from vaccinated quiescent infected to recovered	1/698.75 days	120-1277.5	[8]
*λ_R_*	rate of progression from recovered to susceptible	0	0	N/A
*θ_L_*	ratio of infected progress to clinical symptoms	0.975	0.95-1.0	[8]
*θ_D_*	ratio of infectious that die	0.9	0.8-1.0	[26]
*ε_q_*	efficacy of quarantine (ratio of susceptible successfully quarantined)	0.5	0.1-0.9	[22]
*ε_vs_*	efficacy of vaccine for susceptibles (will move into immune)	0.775	0.6-0.95	[6]
*ε_ve_*	efficacy of vaccine for exposed (latent only)	0.775	0.6-0.95	[5,6]
*ε_c_*	efficacy of culling	0.5	N/A	[10,29]
*ε_s_*	efficacy of short-range movement control	0.5	0.1-0.9	N/A
*ε_l_*	efficacy of long-range movement control	0.5	0.1-0.9	N/A
*T_l_*	time after detection until inter-state movement restricted	6.5 days	1-14	N/A

**Table 3:** Model parameter description and disease input ranges used with supportive references

	Parameter description	Baseline	Range	**Ref**.
*T_v1_*	time after first detection in U.S. until vaccine widely available	33.5 days	7-60	N/A
*T_v2_*	time after further detection locally until vaccine available	17 days	N/A	N/A
*T_q_*	time after detection until quarantine implemented	2 days	1-3	N/A
*T_c_*	time after detection until culling implemented	2 days	1-3	N/A
*η*	number of infected animals needed to trigger official detection	50	N/A	N/A
*k*	constant of proportionality for long-range movement kernel	0.001	N/A	N/A

### Intra-county model

We begin with the micro-scale intra-county model in which deterministic equations modeling disease spread within a county are solved for parameters sampled randomly from across their ranges. First, we assumed that there is no natural death of hosts, so that animals in the model die due to infection or culling. For this case study, the "types" of animals are beef cattle, dairy cattle, cattle on feed, sheep and goats, and pigs. We will refer to each of the susceptible, infectious, recovered, dead, vaccinated, quarantined, etc compartments as a disease stage. Within each county there is no heterogeneity for livestock distributions in respect to the number of farms accounting for the number of animals. Each susceptible host of type *i *in county *x*, denoted *S_i_^x^*, has a certain probability, namely μ*_ij_^mn^*, of becoming infected with the pathogen due to contact with another infected animal of type *j*. This probability is based on the susceptibility to disease of animal type *i *in disease stage *m*, denoted *s_i_^m^*, the infectivity of animal type *j *in stage *n*, denoted ι_*j*_^n^, and a scaled contact rate based on the density of farm animals in the county, denoted *e^-r(x)/a ^*where . Here, *N *is the total number of all types of animals in the county, *A *is the area of the county, and *a *is a constant of proportionality referred to as the characteristic length of local spread. The transmission rate, or probability of infection, is

where the *fraction infected *= *n_j _/N *for *n_j _*the number of animals in (infected) stage *n *of type *j *and represents the probability that a contact is with an infected individual. For our case, we then rewrite the transmission probability as

So, the probability of species *i *in stage *m *becoming infected by species *j *in stage *n *is μ_ij_^mn ^= *ι_j_^n^s_i_^m ^e^-r(x)/a ^n_j _= β_ij_^mn ^n_j _*where *e^-r(x)/a ^*is the true contact rate scaled by the total number of animals, *N*. Also note that for very low densities, *e^-r(x)/a ^*behaves linearly and, as density increases, e^*-r(x)/a *^approaches 1 as its slope approaches zero. The transmission function moves between a linear dependence at low animal density and saturates at high animal density.

**Figure 1:**
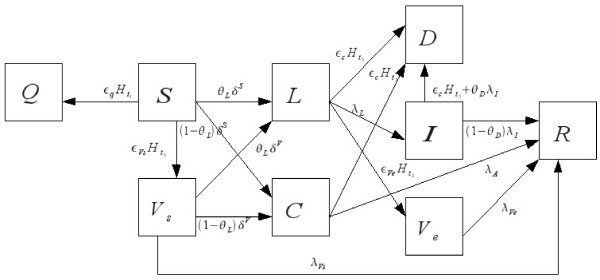
**Description of the intra-county disease progression model**. See Tables 1-3 for specific symbol descriptions used in the model.

The possible progressions through the disease states of our model, which begin in the susceptible state, *S*, and progress to either recovered, *R*, or dead, *D*, are diagramed in Figure 1. After becoming infected, a susceptible host can move into either a subclinical ''latent'' state or a subclinical ''carrier" non-progressing state with probability *θ_L _*or 1 *- θ_L_*, respectively. The host in the subclinical latent (incubation) stage, *L_i_^x^*, with infectivity *ι_i_^L ^*remains for a residence time of *1/λ_L _*upon which the host transitions into a symptomatic infectious stage, *I_i_^x^*. The hosts in the carrier stage, *C_i_^x^*, have infectivity of ι_i_^C ^but never exhibit clinical signs and after a residency time of 1/λ_C _move into a recovered, immune stage, R_i_^x^. We will refer to *L_i_^x ^*and *C_i_^x ^*as the quiescent infected group. Meanwhile, hosts in the infectious stage will have infectivity ι_i_^I ^and remain infectious with time of *1/λ^I ^*after which they will either die or recover with probability *θ_D _*and *1-θ_D_*, respectively. The recovered class remains immune for life.

The intra-county portion of the model also includes mitigation processes such as vaccination, quarantine, and culling, as well as the response time and efficacy of each of these control measures. After 50 hosts are infected in a county, the disease is officially detected with a corresponding time of detection, *τ_d_*, and control measures are implemented with an appropriate time lag. The first response to detection is quarantine. At the time of quarantine, *t_1_*, uninfected hosts are isolated and thus removed from the susceptible compartment. Here, *ε_q _*is the efficacy of the quarantine, so that the total number of animals of type *i *successfully quarantined is *ε_q_S_i_^x^*. The quarantine has a time lag, T_q_, of 1 to 2 days. We used a wide range for between 7 and 60 days when vaccines become widely available after the first time disease is detected in the US and we used an average of 33.5 days to become available locally after subsequent detection of disease in a county. We wanted to test for the impacts of having vaccines ready versus a longer time period for vaccine development. For *ε_Vs _*and *ε_Ve_*, the efficacy of vaccination on susceptible and quiescent infected animals, respectively, the total number of successfully vaccinated animals of type *i *at the time of vaccination, t_2_, is ε_vs _S_i_^x ^+ ε_ve _(L_i_^x ^+ C_i_^x^). There is a lag between time of vaccination and immunity so vaccinated susceptibles are moved into a temporary stage *V_s _*with residency time 1/λ_Vs _and susceptibility to disease reduced by a factor of *r_Vs _*so that β_ij_^VsM ^= *r_Vs _*β_ij_^SM ^where M is one of the infectious states. Similarly, vaccinated latent animals (in L_i_^x^) are moved into stage *V_e _*with residency time 1/λ_Ve _with infectivity reduced by a factor of r_Ve _so that β_ij_^MVe ^= *r_Ve _*β_ij_^ML^. It is assumed that vaccinated carriers exhibit no different behavior than un-vaccinated carriers so that carriers that are vaccinated simply remain in the C_i_^x ^or carrier, stage.

Lastly, we consider culling, which has a lag time of 1 to 2 days after detection and an efficacy of *ε_c_*. Culling can occur in two instances: if a county is under surveillance for the disease, then both infectious and quiescent infected groups are culled at time t_3_*, whereas, if a county is not under official surveillance, then only clinical infectious animals are culled at time *t_3_*. Notice that this implies the ideal situation where no susceptible or recovered animals are culled. A county will be put under surveillance if it is within 20 miles of another known infected county that is under quarantine (this happens if the number of clinical infectious animals in the county is greater than *ν = *50 and enough time, *T_q_*, has elapsed for a quarantine to be put into place) or if the county itself is under quarantine. This surveillance zone estimate is a conservative estimate based on the average surveillance zone size of 30 km for foot and mouth epidemics in Europe. Since accurate pen-side tests for rinderpest are available, good surveillance and methodical separation of infected animals are possible.

The equations for the intra-county model are then

where

and

Finally,  is the set of all times when quarantine occurs in county *x*,  the set of all times when vaccination occurs in county *x*,  when culling occurs in a county *x *not under surveillance, and  the set of all ties when culling occurs in a county *x *under surveillance. For this model, mitigation is conducted on the day scale so that the SIR-type model is run for a full day in a county and at the end of that day mitigation strategies are implemented and numbers of animals in each stage are updated accordingly before running the SIR-type model for the next day.

### Inter-county model

**Figure 2:**
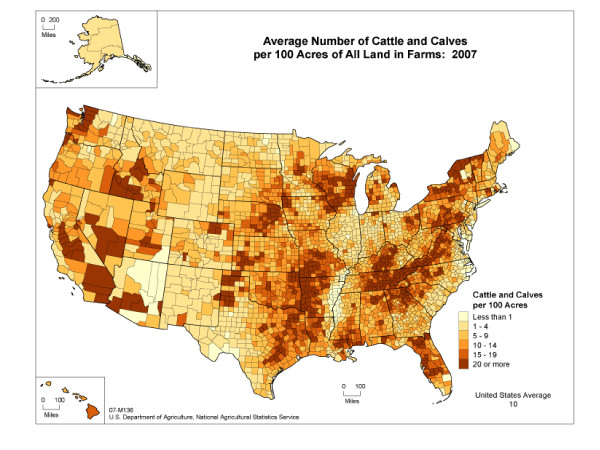
**Density of cattle and calves in the US by county**.

Next, we discuss the macro-scale inter-county and inter-state model. Figure 2 shows the density of cattle in the US with county-level resolution. This and similar data for the other animal classifications are available from the 2007 agricultural census and the cattle are split into beef cattle, dairy cattle, cattle on feed as used in the model [13]. Each susceptible county, *x*, has a probability of becoming infected of *p_x(t) _= 1-e^-Γx(t) ^*where *e^-Γx(t) ^*is the probability of *not *becoming infected and

for *i *the number of species and *y *the number of counties. We use χ to indicate reduced long- or short-range movement due to movement control measures put into place after detection of a disease and use κ as a long- or short-range movement kernel. For this model

κ_s_(x,y) = *e^-(rx-ry)/a ^*and κ_l_(x,y) = *1-e^-kΣi gi(x,y)Δt^*. Here, *a *is a constant of proportionality for short-range movement seen as the length scale of transmission resulting from animal-to-animal contact and fomites, (r_x_-r_y_) is the distance between counties *x *and *y *(on a sphere), *k *is a constant of proportionality for long-range movement, and Δt is the time step being used by the integration scheme. For our simulations, Δt = 0.125 (approximately 1/8 day). Also, *g_i_(x,y) *is the frequency of inter-state movement from state *y *into state *x *for animal type *i *based on data from the US Department of Agriculture [14].

We chose 16 starting locations for the epidemic as case studies for our model. To determine starting locations, we picked two counties from the top ten counties for number of each of the groups of animals we considered (dairy cattle, feedlot cattle, beef cattle, sheep, and pigs). In addition, we started the epidemics in each of the different animal groups to add variation and less predictability to the scenarios. There were several counties with high populations for multiple groups so we minimized duplication by choosing from among the top ten. We also chose several counties (in Florida, Arizona, California, and Wyoming) that have much livestock but are geographically separated from other counties with significant livestock density or numbers. These isolated counties were chosen in order to see the comparative effects of short and long distance movement and movement control for various regions in the United States.

## Results

We ran our model 400 times for each of 16 starting locations throughout the US, exploring different combinations of the various disease properties and mitigation parameters, as well as simple stochastic variation. The majority of simulation runs each produce more than a ten-fold increase in the number of cases in a few days after the start of the epidemic. A few days later, and at much lower levels, the recovered and dead populations rise, reflecting the high mortality rate of rinderpest in cattle. Shortly after the sharp rise of symptomatic animals, a massive quarantine program appears, and culling of symptomatic animals. The next two months of the epidemic reflect a steady spread of disease to new counties and the subsequent application of quarantine and culling to contain the spread in each new region. Within the model, the duration of quarantine is indefinite, although in reality, the quarantine could be lifted once an effective vaccination program occurs.

**Figure 3:**
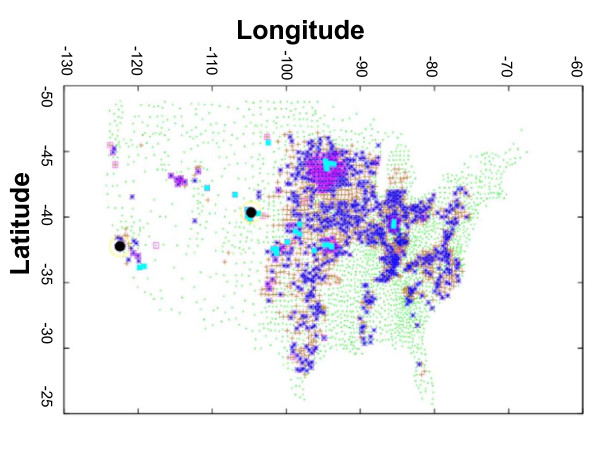
**Geographic progression of one epidemic seeded in Weld County, Colorado**. All counties are shown with green crosses and counties impacted by the epidemic by days 2, 11, 21, 51, and 101 are shown with various symbols.

The spatial-temporal spread of a severe epidemic can be seen in Figure 3, showing the map of the US, colored according to the day each county sees its first case of rinderpest. The epidemic was seeded in Weld County, Colorado, on day 0, and spread to California almost immediately (black circles). By day 11, the disease has already spread to over a dozen locations throughout the US, seeding the second explosion of cases, during days 11 to 16. During the longest phase of the epidemic, from week 3 to 9, nearly all of the 70 million beef cattle in the nation are quarantined, with almost one million beef cattle culled. Rinderpest epidemics spread to essentially every area in the country that contains significant populations of beef cattle. As the rate of the growth of new infections levels off, a great deal of effort and activity is being expended during this portion of the epidemic, as the spread is mitigated by a combination of a quarantine (which reduces the effective reproductive number below one) and the rapid identification and culling of newly symptomatic animals that results from imperfections in the quarantine.

### Sensitivity to model parameters

**Figure 4:**
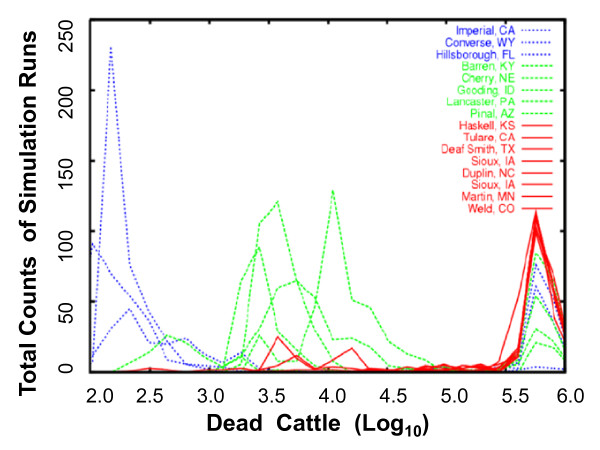
**Consequence realized over 400 runs of varying disease and mitigation parameters for epidemics started at the 16 locations (three groups)**. Counts are the number of simulation runs with the number of total dead cattle.

The worst-case scenario represents only one of many possible instantiations of a rinderpest epidemic (Figure 3). We explored the sensitivity of consequence to variation in nearly all model parameters. Figure 4 illustrates how the total number of dead beef cattle depends on the starting location of the epidemic, as well as the effectiveness of the quarantine. Epidemics were seeded with 100 infected animals of one type (beef cattle, milk cattle, feedlot cattle, swine, or sheep) in one of 16 counties selected to be illustrative of geographic diversity in the epidemiology. Quarantine efficacy was defined to be the fraction of animals protected from infection by the quarantine and was allowed to vary from 0.1 (only a ten percent reduction in infection) to 0.9, representing a ten-fold decrease in the likelihood of disease spread. This parameter involves all possible modes of spread, including animals moving, spread by wildlife, animals being transported, and disease spread with fomites by humans. Considerations such as asymptomatic spread also appear here. The impact of the time between detection of rinderpest in a county and initiation of culling (varied from 1 to 4 days) was nearly as large as that of quarantine efficacy, but most model parameters had a smaller impact on the overall number of animals infected by the epidemic.

The normalized forward sensitivity index of a variable to a parameter is the ratio of the relative change in the variable to the relative change in the parameter. Since all variables depend on many nodes in the network of counties and probability of infection is stochastic, the sensitivity indices were computed numerically based on the mean of approximately 5 000 runs starting in 16 different locations. We computed the sensitivity of total number of animals infected to the disease-related parameters. We found that the total number of infected animals increases with the fraction of animals that progress to symptoms, with the fraction of infected animals that die, and with an increase in the incubation period. The number of infected animals is not very sensitive to intrinsic disease parameters over the range they were varied (reflecting plausible values for these parameters). We varied each of these parameters along their range for 16 different starting locations. We then computed the average number of infected animals across the range of each parameter for the 5 000 runs. The slope of the best fit line for each parameter versus the average number of infected animals was used to calculate the sensitivity index. See Table 4, of sensitivity indices.

**Table 4:** Sensitivity analysis for each varied parameter for the simulations

Parameter	Normalized Forward Sensitivity Index
(ratio infected cows that progress to symptoms)	11.3
(ratio infected sheep that progress to symptoms)	3.4
*θ_D_* (ratio infected that die)	3.4
(ratio infected hogs that progress to symptoms)	0.5
*T_c _*(time after detection until culling implemented)	0.4
*ε_s _*(efficacy short range movement control)	0.3
1/*λ_L _*(residency time in latent stage)	0.2
1/*λ_Vs _*(residency time in vaccinated susceptible)	-0.2
*ε_l _*(efficacy long range movement control)	0.2
*ε_q_*(efficacy of quarantine)	-0.1
1/*λ_I _*(residency time in infectious stage)	0.1
1/*λ_C _*and 1/*λ_Ve _*(time in carrier stage either vaccinated or not)	0.1
*ε_Vs _*(efficacy vaccine for susceptible animals)	0
*T_v1 _*(time after 1^st ^infection until vaccination)	0
*T_l _*(time after detection until long range movement control implemented)	0
*T_q _*(time after detection until quarantine)	0

Rinderpest can be controlled with several mitigation strategies. We use sensitivity analysis to quantify the relative impact of various mitigation strategies on the total number of infected cattle. We found that movement control is not very effective in controlling both variables. Culling, on the other hand, is very effective, especially if implemented promptly [15]. Vaccination can be effective for controlling the size of an epidemic, but only if the vaccine is readily available and stockpiled, which is not currently the case in the US. The last important variable for controlling the epidemic is the time until the epidemic is detected.

### Importance of geography

The most striking find was dependence of the overall epidemic size on the starting location (Figure 4). Overall epidemic size, measured by the number of infected animals for the epidemics started in 16 locations throughout the US, was related to the seed location. Epidemics from the 16 seed locations can be classified according to overall size into small epidemics of 100 to 300 animals (failed epidemics), epidemics infecting 3 000 to 30 000 animals (medium epidemics), and the large epidemics infecting around one million beef cattle. Epidemics infecting 1 000 beef cattle or 100 000 beef cattle rarely occur, although several locations readily produce both failed and large epidemics.

### Geographic flow of infection

From the simulated data, clustering exists around small and very large epidemics with few cases falling between the two extremes. The conditions under which rinderpest reaches large epidemic levels are related to the origin of the disease and whether or not the disease moves into certain key counties in high-livestock-density areas of the US. We have indicated the starting locations of the failed, medium, and large epidemics with appropriately colored symbols in Figure 2 of the density of beef cattle. Further examination of the simulation results indicate that the large epidemics passed through the Midwest at some point early in the epidemic.

The variation in spatial origin and size of observed epidemics suggests further examination of the dependence of the epidemic size on response time and effectiveness of movement controls. Because the parameter values were sampled from a uniform distribution, it is evident that failed epidemics are significantly more likely to occur in the presence of reduced movement of animals. Equally evident, however, is that movement controls alone are not particularly helpful. Clearly, if movement controls prevent all movement, the epidemic would, by definition, not spread. Our model is merely highlighting that single cases can, quite frequently, get through even stringent movement control schemes.

## Discussion

Determining parameter values for rinderpest is difficult in many cases because there is a paucity of spatial historical data and rinderpest has never been present in the US. We can be relatively confident of disease progression parameters within individual hosts, such as the incubation and infectious periods, as well as death rates experimentally [15,16], although exploration of ranges for these parameters is clearly prudent.

The epidemiological parameters are somewhat more difficult to quantify. The most reliable indicator is the historical data of the frequency and size of epidemics. In extrapolating the transmission likelihood from historical data, three significant sources of uncertainty must be lumped together. First, are the intrinsic transmissibility of the disease and susceptibility of animals to the virus, which are likely to be higher than past epidemics because of the long-term absence of circulating rinderpest. Second, are the greatly increased size, density, and transport of livestock in the US. Finally, modern agricultural practices are more highly refined than they were when rinderpest last circulated freely, presumably resulting in better control of infectious disease in general. In order to validate the model for transmissibility parameters, we compare qualitative spatial results with what is known from previous outbreaks of rinderpest in näive herds and with well known recent outbreaks of foot-and-mouth disease. It is important to realize that we are not primarily concerned here with computing the median consequence value for a rinderpest epidemic (that would require quite careful examination of the above three effects). Instead, we aim to explore and quantify the relationship between disease properties, geography, and mitigation strategies to better understand and mitigate the spread of infectious diseases in multi-host populations.

We found that rinderpest spread as expected when started from different geographic locations in the US. For example, in recent foot-and-mouth disease studies it has been shown that number of animals is important in the initial stages of the disease, while density of animals becomes important after the first one to two generations [7,17]. We would expect then that rinderpest requires a path through densely populated areas and an initially large population of livestock in order to spread widely. This was indeed how the model behaved. For instance, an epidemic started in a county in Idaho caused high death rates in that county but was not able to spread to the rest of the US because Idaho is surrounded by states with very low livestock densities. However, an epidemic started in Iowa spread rapidly throughout the high-density belt from the Midwest through eastern Texas. One difficulty in the modern era is that, even if not surrounded by areas with dense populations of livestock, infected animals may be shipped to areas that are densely populated. We also saw that rinderpest spread quickly, which is to be expected from examining the last continent-wide epidemic in näive herds in Africa in the 1890's. Even though transportation was much slower and less widespread, rinderpest spread from the horn of Africa to the tip of South Africa (about 8 000 km) in less than 10 years [11].

Spatial mixing plays an important role in other fast-spreading animal diseases such as foot-and-mouth disease [18], and initial explorations indicate that the same is true for rinderpest. Rinderpest spreads quickly, is highly transmissible, and has a high death rate, so has the potential to burn itself out quickly if enough susceptible animals are not available. Thus, for an initial infection to become an epidemic, rinderpest initially requires a large number of susceptible animals. After the first few generations, high density of hosts is required as with foot-and-mouth disease. So, we assume that rinderpest will only become a large-scale epidemic if it reaches or begins in the high-number, high-density areas in the Midwest of beef cattle in the US. Because of human mobility we not only have to consider proximity but rate of movement of livestock between areas. For example, although California is geographically distant from other high-density livestock areas in the US, high rates of movement between California and the mid to eastern US result in large epidemics with origins in California. We separated the initial locations into three categories: primarily small epidemics, primarily large epidemics, and bimodal distribution of epidemics. In addition, the importance of wildlife in the propagation of rinderpest should not be understated. Although the data on wildlife required to be incorporated into the model are mostly unavailable, wildlife may be an important part of an epidemic.

In all of the simulations, the overall mortality rate never exceeded a few percent, even though the case fatality rate is nearly unity. This is because we concluded that, even in the worst case, ranchers would be able to control the epidemic by identifying and culling the clearly symptomatic animals. The importance of this mitigative strategy is evident in the dependence of the size of the epidemic on both the efficacy and rapidity of quarantine and the rapidity of culling.

The apparent lack of importance of vaccination evident in the sensitivity analysis does not indicate a lack of importance of a highly efficacious vaccine in controlling rinderpest. It simply reflects our expectation that quarantine and culling of the sporadic outbreaks will be utilized to control the epidemic only until the vaccine can be administered and become effective. Such a dependency would show up strongly in a complete economic consequence analysis, which we have not attempted here.

One important advantage to our epidemiological model is its ability to treat multiple hosts on an equal footing. The hosts can differ in either disease progression properties, such as the greatly decreased disease susceptibility of swine to rinderpest, in comparison to cattle. They can also differ in their epidemiological properties, such as the fact that feedlot cattle do not typically return to mingle with beef cattle once they enter the feed lot. Indeed, the low susceptibility of swine to rinderpest is a significant factor in the difference from foot-and-mouth epidemic spread across the US. Although our multi-host model treats the different types of livestock appropriately, we only treated wildlife and the spread of disease by humans (through fomites--humans do not contract rinderpest) implicitly, through the imperfection of both long- and short-range movement restrictions. It will be important to return to these questions in future studies.

The explosive spread of rinderpest apparent in Figures 3 and 4 can be traced to three separate parameters in our model: asymptomatic spread, relatively short incubation times, and a relatively high transmission and susceptibility coefficient. Given the likelihood, even in a naïve outbreak, of a percentage of asymptomatic cases and the possibility of an avirulent strain being introduced and spreading widely, with potential subsequent reversion to virulence, asymptomatic animals play an important role in both the long-term outcome of a rinderpest epidemic and in the best surveillance and mitigation strategies. Here, we focus on the virulent strain of rinderpest to simulate a worst-case scenario for impacts. The ultimate ability to control the disease while losing only a few percent of the Nation's livestock can be traced to the clarity of the signs of disease and the existence of an efficacious vaccine, which led to our assumed rapidity and effectiveness of culling and quarantine.

An important outcome of this study is the importance of geography and the density of susceptible hosts to the spread of rinderpest. The relatively small statistical correlation of epidemic size to movement restrictions in comparison to quarantine and culling should not be interpreted to mean that this control measure is of little importance. There are several lessons learned from these simulations for the management of rinderpest or similar disease outbreak in cattle populations in the US. First, it is far cheaper to implement than quarantine or culling, although the economics of the loss of export are considerable. Second, the impact of preventing the spread to the major cattle populations is a thousand-fold decrease in epidemic size and a significant shortening in the duration of quarantine and culling interventions. Third, the actual effectiveness of movement restrictions depends on several key variables, such as the absolute value of the transmissibility of the virus and the implicit assumptions on the likelihood of spread by fomites or wildlife. Finally, it is impossible to capture the adaptive nature of the mitigative measures in a model such as ours. Our parameter estimates are applied 'for the long haul' and may not reflect potential opportunistic mitigation.

These results strongly support the case for complete eradication of rinderpest. The ability to systematically explore the epidemiology of disease will be important when considering the impacts of climate change and emerging disease, and the robustness of modern agricultural practices. It is also important as a stepping stone to controlling zoonotic diseases and understanding the evolutionary pressures of multi-host pathogens in general. The geography and connectivity of populations plays an important role in the outcome of an epidemic. Using this knowledge of animal population density and connectivity can assist in determining critical populations or locations to apply mitigation or control measures for animal movement.

## Authors' contributions

The study was co-conceived designed by all authors. CM adapted the original code to the Rinderpest model, ran the code for the designed scenarios, performed sensitivity analysis of the parameters, and drafted the manuscript. BMM assisted with the adapted code, and made the figures. JF assisted in model parameterization and manuscript. MH oversaw mathematical accuracy, and assisted in design of figures and analysis. MB calibrated the model to USDA data and other data. MLB wrote the original code for the model and assisted in manuscript preparation. All authors read, amended and approved the final manuscript.

## Competing interests

The authors declare that they have no competing interests.
